# Retrotransposons in the development and progression of amyotrophic lateral sclerosis

**DOI:** 10.1136/jnnp-2018-319210

**Published:** 2018-10-10

**Authors:** Abigail L Savage, Gerald G Schumann, Gerome Breen, Vivien J Bubb, Ammar Al-Chalabi, John P Quinn

**Affiliations:** 1 Department of Molecular and Clinical Pharmacology, Institute of Translational Medicine, University of Liverpool, Liverpool, UK; 2 Division of Medical Biotechnology, Paul-Ehrlich-Institut, Langen, Germany; 3 Social, Genetic, and Developmental Psychiatry Research Centre, King’s College London, London, UK; 4 Department of Basic and Clinical Neuroscience, King’s College London, Maurice Wohl Clinical Neuroscience Institute, London, UK; 5 King’s College Hospital, London, UK

**Keywords:** ALS, genetics

## Abstract

Endogenous retrotransposon sequences constitute approximately 42% of the human genome, and mobilisation of retrotransposons has resulted in rearrangements, duplications, deletions, novel transcripts and the introduction of new regulatory domains throughout the human genome. Both germline and somatic de novo retrotransposition events have been involved in a range of human diseases, and there is emerging evidence for the modulation of retrotransposon activity during the development of specific diseases. Particularly, there is unequivocal consensus that endogenous retrotransposition can occur in neuronal lineages. This review addresses our current knowledge of the different mechanisms through which retrotransposons might influence the development of and predisposition to amyotrophic lateral sclerosis.

## Introduction

Amyotrophic lateral sclerosis (ALS), also known as motor neuron disease, is characterised by the rapid progressive degeneration of the upper and lower motor neurons. This results in the wasting and weakening of the muscles of the limbs and trunk and those involved in speaking, swallowing and facial expression. It is usually fatal within 3–5 years of disease onset, most frequently due to respiratory failure.^1^ In European populations, the incidence of ALS is 2.16 per 100 000 people per year, and the median age at diagnosis is 65.2 and 67 years for men and women, respectively.^2^ The mechanisms underlying the pathogenesis of ALS are not fully understood; however, there have been several hypotheses proposed to explain what drives the development of the disease, which include protein aggregation, oxidative stress, excitotoxicity and defects in RNA processing and axonal transport.^3^ Of the cases of ALS, 5%–10% are reported as familial and the remaining as sporadic. However, this division may not accurately reflect the mechanisms and risk associated with disease development.^S1^ Familial ALS is defined by a positive family history of the disease, but family history may not always be apparent, has varying definitions and many of the mutations identified in cases of familial ALS are also found in those with the sporadic form.^4 S2^ Twin studies estimate a heritability of 60% in sporadic ALS, and first-degree relatives of patients with sporadic ALS have an eightfold increased risk of developing the disease, demonstrating further the importance of genetics in the sporadic disease.^S3 S4^ There have been more than 30 genes associated with familial and sporadic ALS and more genetic risk loci identified through genome-wide association studies (GWAS).^5 6^ The four genes harbouring mutations that cause the greatest number of ALS cases are *SOD1*, *C9orf72*, *FUS* and *TARDBP*, contributing to 47.7% and 5.2% of familial and sporadic cases, respectively, in a recent meta-analysis of European and Asian populations.^7^

The increase in the generation and availability of sequencing data has driven the discovery of new genes involved in the development of ALS, with the rate of discovery doubling every 4 years.^S5^ Despite these findings there is still much to learn about the genetic causes of ALS and how variants affect disease progression. The same mutation in different individuals does not always result in the same clinical progression or even the same disease. The *C9orf72* repeat expansion and mutations in *FUS* and *TARDBP* can lead either to the development of ALS or frontotemporal lobar degeneration (FTLD).^8^ Additional modifying genetic variants and environmental factors or a combination of both are likely to contribute to the phenotypic heterogeneity seen in carriers of these mutations. For example, the intermediate length trinucleotide CAG repeats encoding polyglutamine tracts in the *ATXN2* gene that confer a risk of developing ALS have been associated with *C9orf72* repeat expansion carriers with ALS but not with those carriers with FTLD.^9 10^ Environmental factors have also been implicated in ALS pathogenesis, but it has proven difficult to replicate these experimental outcomes reliably. Proposed environmental risk factors include physical exercise and exposure to heavy metals, pesticides and electromagnetic radiation.^5 11^ Modelling of the population with ALS suggests ALS development is a multistep process requiring six molecular steps, and these are likely to be a combination of genetic and environmental factors.^12^

**Figure 1:**
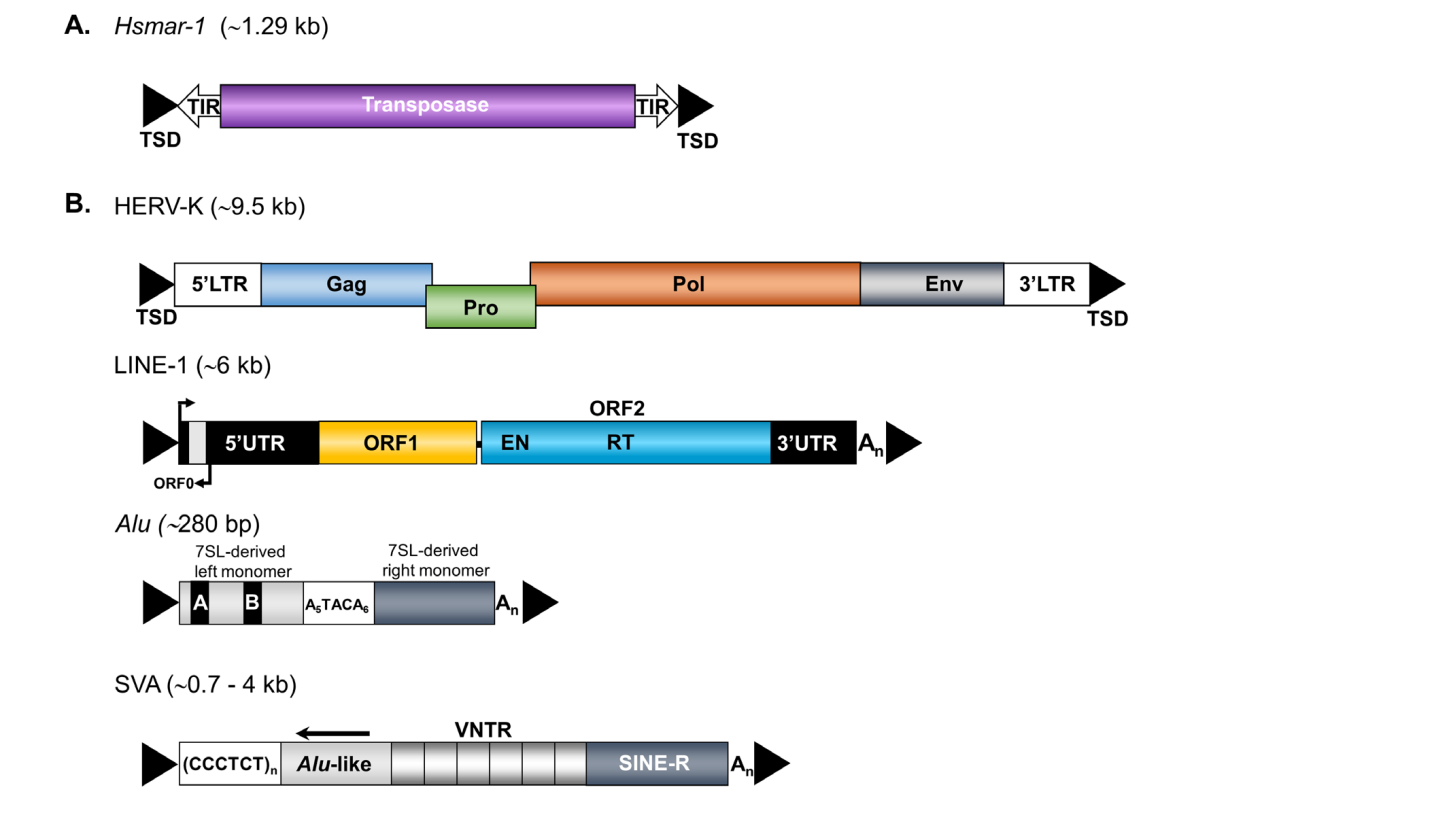
Structure of transposable elements in the human genome. (A) Structure of Hsmar-1, a member of the mariner-like DNA transposon superfamily. The human genome harbours inactive members of seven of the nine eucaryotic superfamilies of DNA transposons.^S33^ (B) Organisation of the retrotransposons HERV-K, LINE-1, *Alu* and SVA. An intact full-length HERV-K provirus is ~9.5 kb long, codes for group-specific antigen (Gag), protease (Pro), polymerase (Pol) and envelope (Env) proteins, and is flanked by ~1 kb LTRs with the 5’LTR including the HERV-K promoter. A functional full-length LINE-1 element is ~6 kb in length, and encodes three open reading frames (ORF0, ORF1 and ORF2), with ORF1 and ORF2 being separated by a 63bp non-coding spacer region. The 5’ untranslated region (5’UTR) harbours the endogenous LINE-1 promoter and an antisense promoter. The 3’ untranslated region (3’UTR) includes the transcriptional termination signal. *Alu* elements are primate-specific SINEs that are ~280–300bp long and are composed of a 7SL RNA-derived left and right monomer separated by an A-rich connector (A_5_TACA_6_) and end in a poly-A tail (A_n_). The SVA element is a composite hominid-specific retrotransposon containing a (CCCTCT)_n_ hexamer repeat, an *Alu*-like region consisting of two antisense *Alu* fragments and an intervening unique sequence, a VNTR region, and a short interspersed element of retroviral origin (SINE-R) region. The length of an intact SVA can vary depending on the number of repeats present in the hexamer and VNTR domains. LINE-1, *Alu* and SVA insertions are characterised by the hallmarks of LINE-1-mediated retrotransposition such as flanking variable TSDs (black triangles), poly-A tails at their 3’ ends (A_n_) and insertion at the consensus target sequence 5’-TTTT/AA-3’. HERV, human endogenous retrovirus; LINE-1, long interspersed nuclear element-1; LTRs, long terminal repeats; SINEs, short interspersed nuclear elements; SVA, SINE-VNTR-*Alu*; TIR, terminal inverted repeat; TSD, target site duplication; VNTR, variable number of tandem repeats.

One component of the human genome that has been largely overlooked in conjunction with neurodegenerative diseases until recently is the group of endogenous transposable elements (TEs), even though such structures contribute to nearly half of our genome. Their role in evolution, disease, regulation of gene expression, response to environmental stimuli and their ability to generate genetic diversity both within a population and within an individual have led to them no longer being considered ‘junk’ DNA but an important part of the human genome.^13–15^ Two classes of TEs can be identified in mammalian genomes:

1. DNA transposons are widely represented in different taxa of organisms including vertebrates. Functional transposons encode a *transposase* protein, flanked by two terminal inverted repeats (figure 1A), and move by a ‘cut-and-paste’ mechanism that involves excising an element and its reinsertion at a new genomic location.^S6^ On insertion, a sequence in the target site is duplicated, generating a target site duplication (TSD).^S7^ DNA transposon sequences account for around 3% of the human genome but, with the exception of at least one family of *PiggyBac* elements in little brown bats,^S8^ no functional DNA transposons have been identified in mammals to date.
2. Retrotransposons comprise approximately 42% of the human genome and propagate via a ‘copy-and-paste’ mechanism, meaning that retrotransposon transcripts are reverse-transcribed into a complementary DNA (cDNA) intermediate which is integrated into a new site of the host genome by retrotransposon-encoded proteins. Retrotransposons can be subdivided into long terminal repeat (LTR) retrotransposons and non-LTR retrotransposons (figure 1B).^16^ This review will bring retrotransposons into sharper focus and discuss how their activity could contribute to the development and progression of ALS.

## LTR retrotransposons

**Figure 2:**
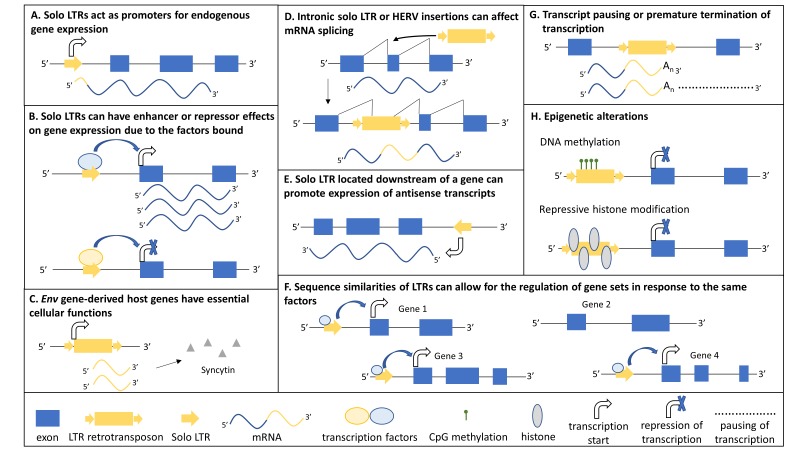
﻿The effects of LTR retrotransposon insertions on the host genome. Both intact full-length HERV proviruses and solo LTRs that are the result of recombination events can affect host gene expression. The mechanisms through which these insertions can act are outlined. (A) LTR sequences contain regulatory domains to control the expression of the proviral HERV insertions and may become exapted as a novel promoter controlling host gene expression often in a tissue-specific manner. (B) The HERV LTRs contain binding sites for transcription factors that can either act to enhance or repress host gene expression depending on the protein complexes bound. (C) A small number of HERV insertions retain coding capacity and specific insertions play an important role in humans; for example, the Env protein of a HERV-W insertion is involved in human placental morphogenesis and the formation of the syncytiotrophoblast. (D) The full-length HERV provirus or solo LTRs in an intron can result in alternative splicing of the host gene due to splice sites present in the HERV sequences. (E) Full-length HERV provirus or solo LTRs located downstream of a host gene can promote the expression of antisense transcripts of this gene. (F) Sequence similarities between HERV LTRs across the genome provide binding sites for the same transcription factors allowing for the regulation of a network of genes. (G) HERV-encoded poly-A signals that are located in introns can cause premature termination or pausing of transcription. (H) DNA methylation and repressive histone modifications (eg, H3K9me3) act to silence HERV expression and can impact on neighbouring gene expression. This figure was compiled from references,^15^ S9 and S34. HERV, human endogenous retrovirus; LTR, long terminal repeat.

Human endogenous retroviruses (HERVs) belong to the class of LTR retrotransposons that constitute approximately 8% of the human genome. They are flanked by LTRs which contain transcriptional regulatory domains controlling expression of the proviral DNA but have also been exapted as regulatory elements for endogenous genes (figure 2).^S9 S10^ Endogenous retroviruses resulted from the repeated infection of germ cells by exogenous retroviruses and became unable to reinfect due to mutations that accumulated in their proviral DNA.^17^ As a consequence, expression of endogenous retrovirus proviral DNA does not lead to infectious particles, and there is currently no evidence that endogenous retroviruses still mobilise in humans, although this cannot be ruled out.^17 18^ The classification of HERVs is complex and dependent on the methodology used, with HERVs divided into 50–200 families.^19^ One of the methods is primarily based on the tRNA that binds to the viral primer binding site which for HERV-K is the lysine (K) tRNA, and the most recent inherited proviral insertions in the human genome are the HERV-K sequences.^17^ A complete autonomous HERV-K sequence harbours 7–9 kb of DNA coding for the viral proteins *gag*, *pro*, *pol* and *env*, and is flanked by ~1000 bp LTRs (figure 1B). There are a small number of complete HERV-K elements in the human genome; however, the majority of HERV families no longer encode functional proteins, and in many cases the only remnant of the HERV integration is a solo LTR due to the removal of the originally LTR-flanked region by recombination events. HERV-K insertions are present in Old World monkeys, apes and humans, and are likely to have entered the primate lineage after the divergence of New and Old World monkeys.^S11^ Similarities in sequence allow for the identification of 10 HERV-K families (HML-1 to HML-10), and a number of HML-2 loci, the youngest family, are human-specific, indicating their continued mobilisation during human evolution.^18^ Recent reports have also identified polymorphic HERV-K insertions for their presence/absence in humans,^20^ suggesting recent mobilisation activity and the possibility that some HERV-K copies might currently still retain the capability to mobilise in present-day humans. Multiple neurological diseases, including multiple sclerosis, schizophrenia and ALS, have been associated with HERVs, and HERV-K is the predominant family studied in the pathogenesis of ALS, but a direct cause–effect relationship has yet to be proven.^21 S12^

## Non-LTR retrotransposons

**Figure 3:**
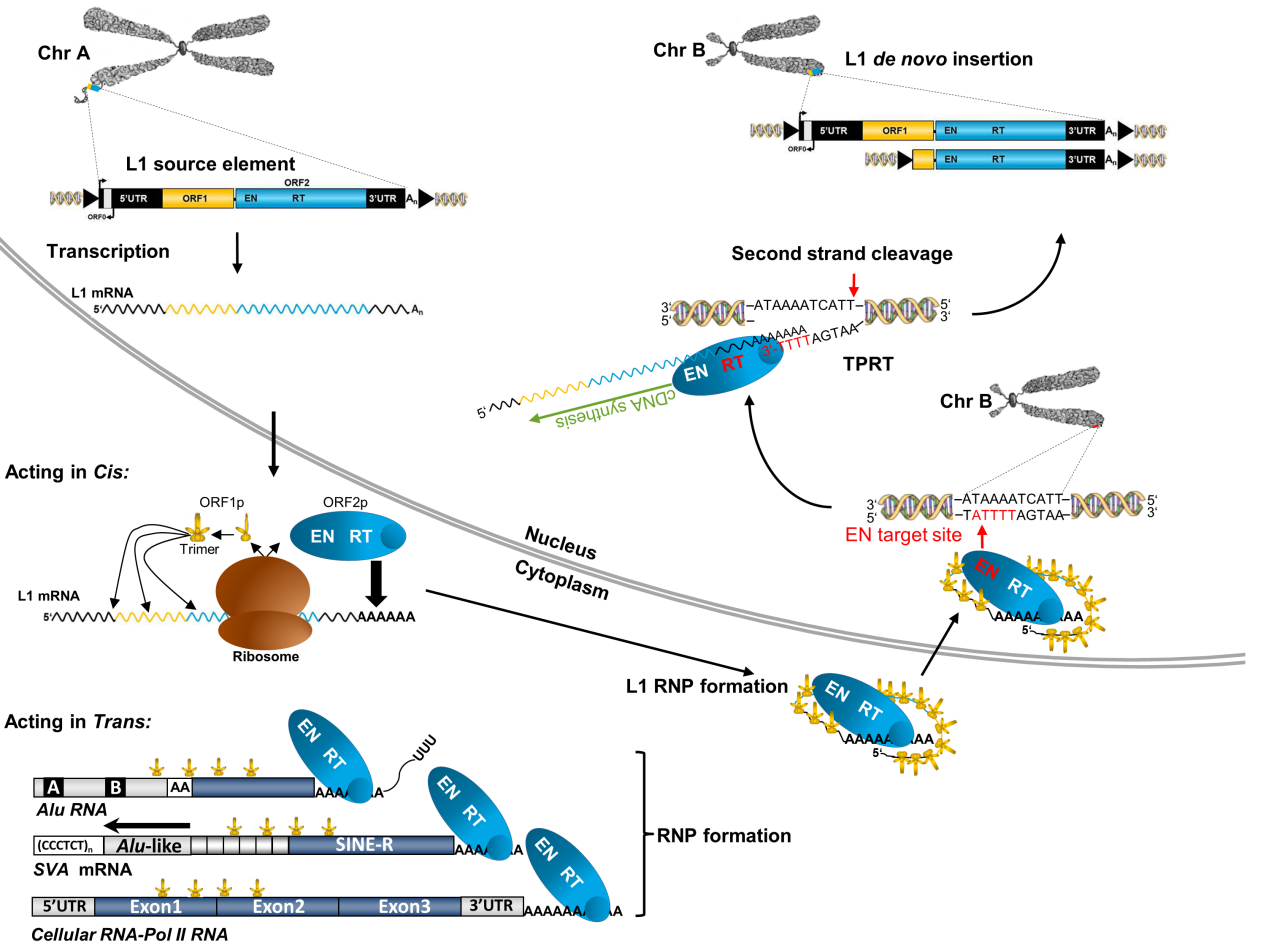
Schematic of the endogenous LINE-1 retrotransposition cycle. An intact, full-length LINE-1 source element on chromosome A is transcribed by RNA polymerase II. The resulting bicistronic mRNA is exported into the cytoplasm where translation of the encoded open reading frames (ORFs) occurs. *Acting* *in cis*: ORF1p and ORF2p exhibit a *cis*-preference for their own mRNA molecule. A multitude of ORF1p trimers and as few as one copy of ORF2p associate with their encoding RNA to form a LINE-1 ribonucleoprotein (L1 RNP) particle. Either the entire RNP or RNP components enter the nucleus where LINE-1 endonuclease (EN) activity nicks the genomic DNA of chromosome B at the LINE-1 consensus 5’-TTTT/A-3’, exposing a 3’hydroxyl residue from which the LINE-1 reverse transcriptase (RT) initiates target-primed reverse transcription (TPRT) of the associated LINE-1 mRNA. Processes leading to second-strand cleavage and second strand-complementary DNA (cDNA) synthesis are still unclear. TPRT results in target site duplication-flanked LINE-1 de novo insertions which are seldom full-length and usually 5’-truncated. *Acting* *in trans*: Occasionally, LINE-1-encoded ORF1p and ORF2p bind *Alu* RNA, SVA mRNA or cellular host gene-encoded RNA polymerase II mRNAs in the cytoplasm, form RNPs with these RNAs and mediate their *trans*-mobilisation by TPRT. The figure has been adapted from multiple reviews (refs 31 S18 S35) (ORF1p, protein encoded by ORF1 of LINE-1; ORF2p, protein encoded by ORF2 of LINE-1). LINE-1, long interspersed nuclear element-1; SVA, SINE-VNTR-*Alu*; UTR, untranslated region; VNTR, variable number tandem repeat.

**Figure 4:**
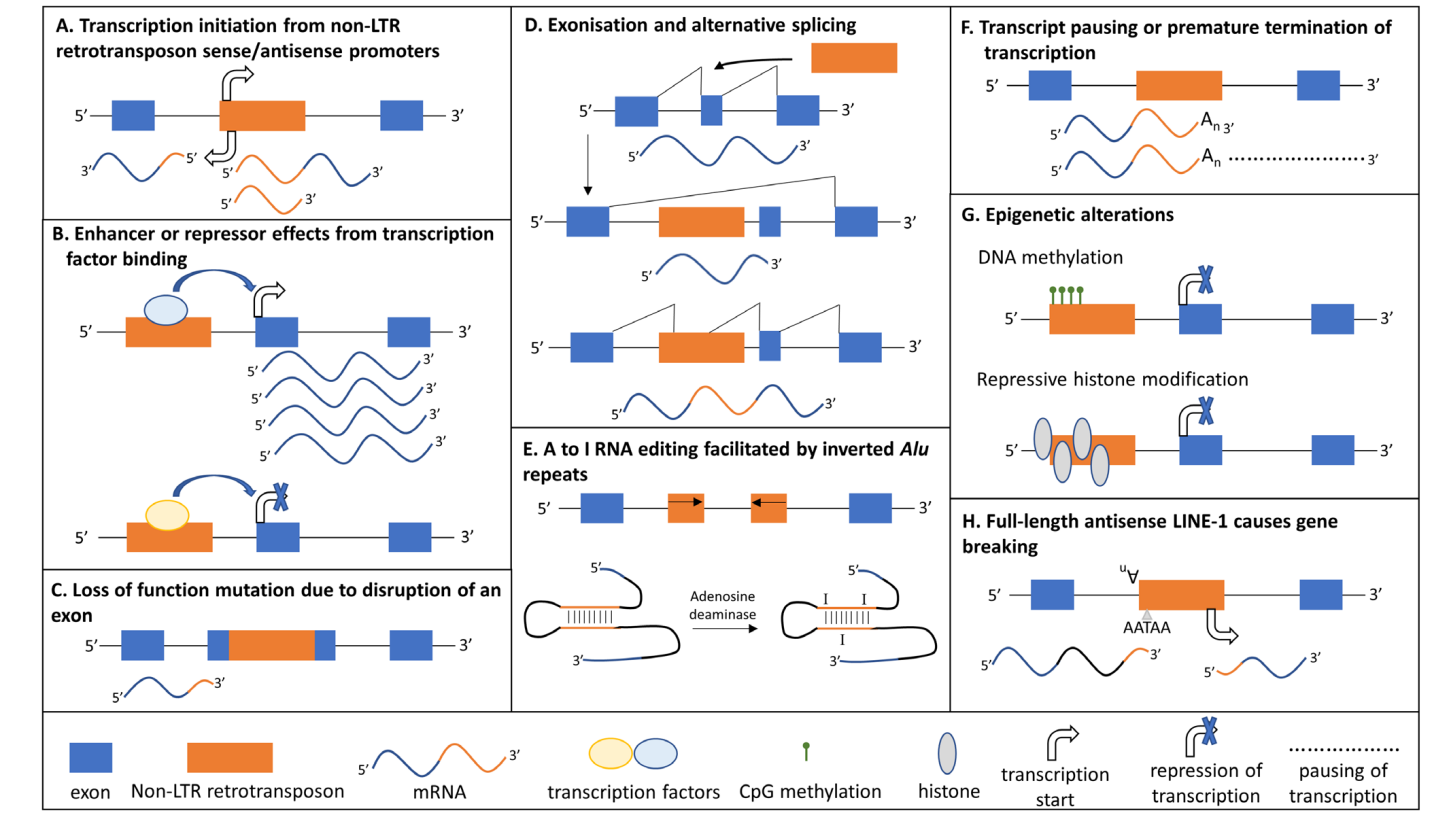
The effects of non-LTR retrotransposon insertions on host gene expression. Non-LTR retrotransposon insertions can affect gene expression through multiple mechanisms and the major mechanisms are outlined. (A) The control of host gene expression by sense and/or antisense promoters of neighbouring non-LTR retrotransposon insertions has been reported for LINE-1 and *Alu* elements,^16^ and the initial 328 bp of a specific subtype of SVAs has been reported to harbour promoter activity.^S36^ Transcriptional start sites have been identified within LINE-1, *Alu* and SVA sequences.^S37 S38^ (B) LINE-1, *Alu* and SVA sequences contain binding sites for transcription factors that can have either positive or negative regulatory effects on the expression of neighbouring host genes depending on the protein complexes bound.^S39^ (C) LINE-1, *Alu* and SVA insertions into exons can cause loss of function mutations.^15^ (D) Splice sites within LINE-1, *Alu* and SVA insertions residing in introns can result in new exons within host genes and alternative splicing.^15^ (E) When two *Alu* repeats are inserted in the opposite orientation in close proximity, base pairing between the two repeats can occur in the mRNA forming double-stranded RNA. Adenosine deaminases can bind to the double-stranded RNA and deaminate adenosine to inosine (A to I editing) affecting transcript stability. As inosine is recognised by the translational and splicing machinery as guanosine, this RNA editing could lead to an amino acid substitution (if it occurs in the coding sequence), alternative splicing or modification of microRNA binding.^S40^ (F) The adenosine-rich nature of LINE-1, *Alu* and SVA transcripts can introduce premature polyadenylation and/or RNA polymerase II transcriptional pause sites into genes, thereby resulting in termination of transcription within the retrotransposons' sequence or reducing their expression.^S38 S41^ (G) Epigenetic alterations at the integration site of a new retrotransposon insertion can restrict retrotransposon expression and include DNA methylation (LINE-1, *Alu* and SVAs contain multiple CpG sites) and heterochromatin formation which can also lead to the repression of neighbouring genes.^53^ (H) Full-length LINE-1 insertions that insert in the antisense orientation into an intron of a cellular gene can split the gene’s transcript into two smaller transcripts through a mechanism known as gene breaking.^S42^ LINE-1, long interspersed nuclear element-1; LTR, long terminal repeat; SINE, short interspersed nuclear elements; SVA, SINE-VNTR-*Alu*; VNTR, variable number tandem repeat.

Non-LTR retrotransposons lack LTRs, include the families of long interspersed nuclear element-1 (LINE-1/L1), short interspersed nuclear elements (SINEs) and SINE-VNTR-*Alu* (SVA) retrotransposons in humans (figure 1B), and represent the only group of TEs that is still currently mobilised in the human genome.^16^ The LINE-1 subfamily represents the only autonomous group of human non-LTR retrotransposons because members of this subfamily encode those proteins that are essential for their mobilisation and there are 80–100 retrotransposition competent LINE-1 elements in the human reference genome.^22^ A functional, full-length LINE-1 element is ~6 kb in length, contains a 5’ and 3’-untranslated region (UTR), three open reading frames (ORF0, ORF1, ORF2) whereby ORF1 and ORF2 are separated by a 63bp spacer region, a poly A-tail at its 3’ end, and is flanked by variable length TSDs (figure 1B). The 5’ UTR includes a sense and antisense promoter. ORF1p is a ~40kDa protein with RNA binding and chaperone activities.^S13^ ORF2p has a molecular weight of ~150kDa and harbours endonuclease (EN)^S14^ and reverse transcriptase (RT) activities^23^ (figure 1B). Both ORF1p and ORF2p are essential for retrotransposition of their own mRNA in cis which is performed by a mechanism termed target-primed reverse transcription (figure 3). This mechanism involves the nicking of the bottom strand of the target DNA by the EN domain of ORF2p to expose a 3’hydroxyl for priming reverse transcription of LINE-1 mRNA by the RT domain of ORF2p.^S15^ Due to the presence of a conserved antisense promoter in full-length LINE-1 elements,^S16^ a third ORF named ORF0 has recently been identified in selected human LINE-1 elements with ORF0p enhancing their retrotransposition.^S17^ The non-autonomous non-LTR retrotransposons *Alu* and SVA do not have any protein-coding capacity and recruit the LINE-1 protein machinery for their own mobilisation in trans. *Alu* elements are primate-specific SINEs derived from the 7SL RNA gene, and there are approximately 1.5 million copies in the human genome (figure 1B).^24^ SVAs are hominid-specific composite elements consisting of a 5’ hexamer repeat that can be variable in length ((CCCTCT)_n_), two *Alu* fragments in antisense orientation, a GC-rich variable number tandem repeat (VNTR), a SINE-R sequence derived from an HERV-K10 element and a poly-A tail following a polyadenylation signal (figure 1B).^25^ The continuing expansion of non-LTR retrotransposons has contributed to more than a third of the human genome shaping its organisation and gene expression through a variety of mechanisms, including insertional mutagenesis, deletions at the insertion site, 5’- and 3’ transductions, non-allelic homologous recombination, transcript pausing or termination, antisense/sense promoter effects, alternative splicing, heterochromatisation and processed pseudogene formation.^13 16^ Furthermore, non-LTR retrotransposon insertions can affect host gene expression by numerous mechanisms (figure 4).

It is well established that new LINE-1, *Alu* and SVA mobilisation events occur in most instances during early embryogenesis and to some extent in germ cells.^26^ The neuronal lineages in hippocampus, cerebellum, caudate nucleus and cortex (reviewed in ref 26) support mobilisation of human endogenous LINE-1 elements, and LINE-1-mediated insertions occur throughout fetal and adult neurogenesis as well as in mature neurons, resulting in somatic mosaicism in the adult brain.^27–31^ LINE-1 retrotransposition has also been demonstrated in tumour cells, resulting in somatically acquired insertions in cancer genomes (reviewed in S18). De novo retrotransposition events that occur in germ cells and during early embryonic development prior to partitioning of the germline are heritable and create genetic variation in the population, whereas those occurring in somatic tissues will not be passed on to the next generation but could influence cellular function and fitness of the individual.

## Detection of reverse transcriptase activity in patients with ALS

It has been hypothesised that retroviral activity may, at least in part, be involved in ALS development.^32^ Evidence for this hypothesis includes motor neuron dysfunction in mice caused by a retroviral infection, the identification of antibodies in the sera of patients with ALS directed against retroviral proteins of the human T-lymphotrophic virus and the development of ALS-like syndromes in patients infected with the human T-lymphotrophic virus-1 and HIV.^32 S19 S20^ Also, patients with HIV-associated ALS syndrome may experience improvement in such symptoms with antiretroviral treatment.^33 34^ Both retroviruses and autonomous retrotransposons encode an RT enzyme generating a cDNA copy of the proviral and retrotransposon-encoded mRNA, respectively.^17 23^ RT activity was detected in 59% of sera from patients with ALS, but only in 5% of sera from an unaffected control group, but it could not be attributed to the presence of any known human exogenous retrovirus.^35^ A second study also detected a significant difference in RT activity in the sera of patients with ALS compared with non-blood relative controls (spouses and healthy volunteers) (47% vs 18%); however, there was only a small difference in RT activity when comparing patients with ALS with blood relative controls (first degree, aunt or cousin) (47% vs 43%).^36^ Comparable levels of RT activity in blood relative controls of patients with ALS and the absence of any detectable exogenous retrovirus suggest that the origin of the RT activity is endogenous. A recent study demonstrating the activation of an endogenous retrovirus in at least a subpopulation of patients with sporadic ALS^37^ suggests that this endogenous RT is at least one source of the detected RT activity. Determining any potential role of RT in the pathogenesis of the disease is hugely important to improve our understanding of the development of ALS. If the increase in RT activity in ALS was shown to have a causative role in the pathogenesis of the disease, well-established RT inhibitors may provide a viable target for treatment.

## Global changes to TE regulation in ALS

There is evidence for a global activation of TEs in ALS, which includes both LTR and non-LTR retrotransposons, and TEs belonging to both retrotransposon, and DNA transposon classes are regulated by TAR DNA-binding protein 43 (TDP-43), a protein involved in neurodegenerative disorders including ALS, FTLD and Alzheimer’s disease.^8^ ^S21^ As previously stated, mutations in the *TARDBP* gene encoding the TDP-43 protein have been identified in both familial and sporadic cases of ALS and FTLD.^7^ Moreover, the accumulation of TDP-43 in the cytoplasm occurs in the majority of ALS and 45%–60% of FTLD cases.^S22 S23^ TDP-43 is a nucleic acid binding protein with distinct binding affinities for RNA and DNA and is involved in the regulation of transcription and splicing, miRNA processing, and the stability and transport of mRNA.^S24^ One study found that transcripts derived from LTR and non-LTR retrotransposons and DNA transposons are targets of TDP-43 in humans, rats and mice, and that this association of TDP-43 and TE transcripts is decreased in the brain tissue of patients with FTLD compared with controls.^38^ This study also demonstrated that in two different mouse models of TDP-43 dysfunction (overexpression and striatal depletion), TE transcripts were overexpressed; the authors hypothesised that TE overexpression may be part of TDP-43 pathology and contribute to TDP-43-related neurodegeneration. Another model system of ALS using *Drosophila melanogaster*, in which the expression of human TDP-43 in either neurons or glia resulted in protein aggregation, motor impairment and premature death, was used to investigate the relationship of TDP-43 and TEs further.^39^ Human TDP-43 overexpression was found to be correlated with elevated expression of members of the class of LTR retrotransposons and LINE families in *Drosophila* due to the loss of small interfering RNAs that act post-transcriptionally to repress these elements. Further, they found that the expression of human TDP-43 and activation of retrotransposon expression caused cell death through DNA damage-mediated signalling. It was shown that this type of toxicity in glial cells was due to the activity of the endogenous retrovirus *gypsy* as the phenotype could be rescued when *gypsy* expression was inhibited either genetically or pharmacologically (RT inhibitors). The data suggest that the activation of retrotransposons could be contributing to TDP-43-mediated neurodegeneration, which includes ALS and FTLD. Similarly, a study analysing the transcriptome of the frontal cortex and cerebellum of patients with ALS demonstrating *C9orf72* GGGGCC repeat expansions, identified a significant increase in transcripts from both classes of TEs (retrotransposons and DNA transposons) in the frontal cortex when compared with controls.^40^ Although *C9orf72* expansion-negative patients with ALS also demonstrated a similar trend of increased TE expression in the frontal cortex, this was not statistically significant. The study did not find an association either between TDP-43 levels or pathology and the increase in TE transcripts; however, the changes observed positively correlated with RNA polymerase II activity. The data suggest that there was a global change in TE expression and activity in the brains of certain patients with ALS, and the exact role, if any, of the different TE families in disease progression is still to be determined. Table 1 summarises the findings from these studies linking TE expression to ALS and FTLD. In addition, several other neurological diseases have been associated with retrotransposon activity, more specifically LINE-1, including Rett syndrome, ataxia telangiectasia, autism and schizophrenia,^S25–S29^ and therefore the dysregulation of retrotransposons may be part of the ALS disease process and play a wider role in neurological conditions.

**Table 1:** Summary of studies linking TE activity to ALS and FTLD

Study	Transposable element	Cell line and animals models	Human samples
Andrews *et al* 35﻿	–	–	Reverse transcriptase activity was detected using a product-enhanced reverse transcriptase assay in the sera of a significantly higher number of patients with motor neuron disease than controls (56% vs 5%).
Steele *et al* 36﻿	–	–	Using a product-enhanced reverse transcriptase assay serum reverse transcriptase activity was detected in a higher proportion of patients with sporadic ALS (47%) compared with unrelated controls (18%) but not compared with blood relatives of patients with ALS (43%).
Douville *et al* 41﻿	LTR retrotransposon (HERV-K)	–	HERV-K *pol* transcript expression was significantly increased in the brains of patients with ALS compared with controls as was neuronal HERV-K reverse transcriptase protein expression. Specific genomic loci from which the HERV-K transcripts originated were identified and included a locus unique to the ALS samples. In ALS brain tissue reverse transcriptase expression was positively correlated with TDP-43 and the reverse transcriptase protein colocalised with TDP-43.
Li *et al* 38	LTR and non-LTR retrotransposons, DNA transposons	TE transcripts are targeted by TDP-43 in both rats and mice. TE transcripts are overexpressed in two different models of TDP-43 dysfunction (overexpression of human TDP-43 in transgenic mice and depletion of TDP-43 in mice striatum).	TDP-43 targets TE transcripts in the human brain and this binding is significantly reduced in FTLD brain tissue compared with healthy controls.
Li *et al* 37	LTR retrotransposon (HERV-K)	The expression of the HERV-K Env protein in human neurons caused a decrease in cell number and retraction of neurites and could contribute to neurotoxicity. HERV-K expression was activated in human neurons on the transfection of TDP-43, and TDP-43 was shown to bind to the LTR of the HERV-K. Transgenic mice expressing HERV-K *env* gene in neurons showed a specific loss of upper and lower motor neurons, neuronal DNA damage and motor dysfunction.	Expression of the HERV-K *gag*, *pol* and *env* transcripts and the Env protein was detected in the cortical and spinal neurons of patients with ALS but not in those of healthy controls.
Krug *et al* 39﻿	LTR and non-LTR retrotransposons	Neuronal and glial overexpression of human TDP-43 in *Drosophila* resulted in a loss of small interfering RNAs that repress retrotransposon expression, and therefore the levels of both LTR and non-LTR retrotransposons were elevated. The toxicity associated with the overexpression of the gypsy ERV element was recused by either blocking its expression or by using reverse transcriptase inhibitors.	–
Prudencio *et al* 40	LTR and non-LTR retrotransposons, DNA transposons	–	TE expression was significantly increased in the frontal cortex of C9orf72-positive patients with ALS compared with individuals who were C9orf72-negative and with healthy controls. The increase in expression of TE transcripts was not correlated with TDP-43 transcript levels or the levels of phosphorylated TDP-43.
Mayer *et al* 42﻿	LTR retrotransposon (HERV-K)	–	HERV-K transcript levels were detected at relatively high levels from 24 different loci across brain and spinal cord samples, and no significant difference was observed between ALS and controls. In addition, full-length Env protein could not be detected in the ALS and control samples; however, there were data indicating the presence of truncated protein variants in the ALS samples.

ALS, amyotrophic lateral sclerosis; FTLD, frontotemporal lobar degeneration; HERV, human endogenous retrovirus; LTR, long terminal repeat; TDP-43, TAR DNA-binding protein 43; TE, transposable elements.

## Activation of endogenous retroviral expression in ALS

Several studies have been carried out, outlined below, implicating a role of HERV-K-derived gene products in the pathogenesis of ALS. There is a significant increase in HERV-K *pol* expression in cortical brain tissue from patients with ALS compared with non-ALS controls.^41^ HERV-K *pol* levels from the HML-2 and HML-3 subfamilies differ across brain regions in patients with ALS but not in the non-ALS controls, as do the genomic loci where the transcripts originate, suggesting unique patterns of expression in the brains of those with ALS. Immunohistochemistry shows the presence of HERV-K RT protein is limited to neurons and at significantly higher levels in patients with ALS than in controls.^41^ In a subsequent study, transcripts of the *gag*, *pol* and *env* HERV-K genes were elevated in the brain tissue from patients with ALS over controls, and expression of the HERV-K HML-2 Env protein was detected in the cortical and spinal neurons of patients with ALS but not in normal or Alzheimer’s disease brain tissue.^37^ Although this provides evidence for the presence of HERV-K expression in ALS, it is not clear if this is part of the pathogenic process or a consequence of it. To elucidate the potential role of HERV-K in the pathogenesis of ALS, a transgenic mouse line expressing the HERV-K HML-2 Env protein in neurons has been generated.^37^ The transgenic animals develop progressive motor neuron degeneration involving both the upper and lower motor neurons, atrophy of the motor cortex, DNA damage and morphological abnormalities of the neurons resulting in a 50% mortality rate by 10 months, suggesting that HERV-K expression is a cause rather than a consequence. Additionally, transfection of the HERV-K HML-2 genome or HERV-K HML-2 *env* gene alone into human neuronal cells causes a decrease in cell number and retraction of neurites.^37^ In contrast to the above studies, a recent report did not detect any compelling differences in the transcription of HERV-K HML-2 loci in ALS versus control brain and spinal cord samples and was unable to confirm the presence of full-length HML-2 Env protein in ALS and control samples.^42^ Table 1 summarises the data from the above studies.

A recent report on five patients with HIV with motor neuron disease analysed the HERV-K RNA expression level in response to antiretroviral therapy.^33^ Data on HERV-K RNA levels at the onset of neurological symptoms were available for two patients who showed elevated HERV-K expression levels in plasma that became undetectable after treatment with antiretroviral therapy.^33^ HIV-1 infection is known to induce HERV-K expression, so it is unclear whether the antiretroviral therapy directly inhibits HERV-K or the reduction in HERV-K RNA is due to a loss of activation by HIV-1.^S30^ Clinical trials are currently under way testing the ability of antiretroviral therapy to suppress HERV-K expression and the safety and tolerability of these drugs in patients with ALS (NCT02437110, NCT02868580 and Motor Neurone Disease Australia’s The Lighthouse Project). The results of these trials will be important in determining if the potentially pathogenic expression of this element could be modified with existing drugs.

## Germline retrotransposon insertion polymorphisms

De novo retrotransposition events are detectable in approximately 1 of 20 live births for *Alu* elements, 1 of 150 for LINE-1 and 1 of 1000 for SVA elements, and within the global human population there are an estimated 392 million private insertions unique to those harbouring them.^14^ It is estimated that every human has on average 180 LINE-1, 1283 *Alu* and 56 SVA presence/absence insertion polymorphisms,^43^ which are an important source of genetic variation. Genetic variants generated by the presence or absence of these retrotransposons are termed retrotransposon insertion polymorphisms (RIPs). Due to the complex nature of the analysis, this type of genetic variation is not routinely studied in large cohorts, although there are a wide range of bioinformatics tools available to analyse such variation.^44^ To date, no actively retrotransposing HERV elements have been reported to be able to create new insertions in the human genome. However, 36 polymorphic HERV-K insertions have been identified that are not present in the human reference genome in a study analysing over 2500 genomes.^20^ The few polymorphic HERV-K insertions are therefore a much smaller source of genetic variation compared with the huge number of polymorphic non-LTR retrotransposon insertions. To date, 124 different LINE-1-mediated insertions have been identified as the genetic cause of diseases such as hereditary cancer, haemophilia, X linked dystonia parkinsonism and neurofibromatosis type 1.^15^ Mechanisms by which LINE-1-mediated de novo retrotransposition events affect the respective disease-related host genes include gene inactivation through aberrant splicing and exonic insertions causing frameshifts, deletions at the site of insertion and also the incorporation of the element’s sequence into the host protein affecting its function.^15^ Many of these disease-causing events are rare insertions resulting in a robust phenotype, but there are also more common RIPs that have been shown to affect the function of the gene they have inserted into. For example, a SVA-E insertion into intron 8 of the CASP8 gene is associated with transcript abnormalities and with an increased risk of breast cancer but a decreased risk of prostate cancer.^45^ There is also evidence for common *Alu* RIPs as candidate causative variants in diseases such as multiple sclerosis when identified to be in linkage disequilibrium with trait-associated single nucleotide polymorphisms (SNPs) identified through GWAS.^46^ A study into the impact of RIPs on human health and disease through changes in gene expression included a global analysis of LINE-1, *Alu* and SVA RIPs in linkage disequilibrium with SNPs associated with complex diseases from GWAS, and found 2474 such elements in a European population.^47^ This study, using a focus on the human immune system, identified candidate RIPs that could lead to the disease through changes in gene regulation.^47^ LINE-1 RIPs have also been shown to be enriched in intragenic regions and different gene ontologies and pathways when those found in controls were compared with individuals with schizophrenia.^S29^ Analysis of SNP variation demonstrated there is a 14.3% genetic correlation between ALS and schizophrenia, suggesting convergent biological mechanisms between the two diseases.^S31^ The burden of RIPs within an individual will vary, and therefore the genes and pathways affected by their presence could also differ.

There are data available on those RIPs that have already been identified, collated in a review^44^ and in databases such as the European database of L1-HS retrotransposon insertion in humans (euL1db.unice.fr). There have also been large-scale studies addressing RIPs in the 1000 genomes data.^S32^ So far, this list comprises approximately 40 000 *Alu*, LINE-1 and SVA RIPs in the human populations studied, but it will grow as further genomes are analysed. This list of RIPs includes several that are present in genes and genetic loci that are associated with ALS. It is possible that these retrotransposon insertions affect splicing and expression of the gene where they inserted through the introduction of novel splice sites and regulatory domains (figure 4 for major mechanisms).^15 47^ We propose that these RIPs and others that have yet to be identified have the potential to alter the function or expression of the genes they have inserted into and could act as genetic predisposing factors for ALS. Extensive analysis of those pathways containing genes potentially affected by RIPs is beyond the scope of a review. However as an example the NEK1 locus, a gene recently identified to contain risk variants for ALS and involved in mitochondrial function and DNA damage response,^48^ has both reference non-LTR retrotransposon insertions and RIPs that could impact on the regulation and function of this gene. To explore this hypothesis, RIPs would need to be addressed in patients with ALS and controls to determine if there are insertions that occur more frequently in one population compared with the other. The functional consequences of any insertions identified that are associated with disease risk would need to be investigated further. One such experiment could involve developing a cell culture model of induced pluripotent stem cells from patients harbouring the insertion, which could then be removed using CRISPR to evaluate the consequences of the specific insertion on host gene expression.

## Somatic mobilisation of non-LTR retrotransposons in the brain

Non-LTR retrotransposons can affect cellular function through insertions in the germline but also via their mobilisation in adult tissues, which includes neuronal cells. The evidence for the mobilisation of non-LTR retrotransposons in neuronal cells has come from a combination of studies using cell lines, animal models and human tissue. By applying engineered LINE-1 retrotransposition reporter elements in cell culture assays, it was demonstrated that rat neuronal progenitor cells, human fetal brain neuronal progenitor cells, neuronal progenitor cells derived from human embryonic stem cells and mature non-dividing neurons can support human LINE-1 retrotransposition in vitro.^28–30^ In addition, an enhanced green fluorescent protein marked human LINE-1 retrotransposition reporter transgene in mice resulted in somatic mosaicism in the brain.^30^ A quantitative multiplexed PCR assay to determine the endogenous LINE-1 copy number in a given genome demonstrated that there is an increase in LINE-1 copies in several brain regions compared with the heart and liver from the same human individual, with the highest number found in the hippocampus.^28^ Using a technique termed retrotransposon capture sequencing, endogenous somatic retrotransposition events in the human brain were identified and characterised.^49 50^ Retrotransposon capture sequencing was used to generate libraries of retrotransposon insertions in genomic DNA from the hippocampus and caudate nucleus of three donors of advanced age (average 92 years old). Subsequent next-generation sequencing identified 7743 LINE-1, 13 692 *Alu* and 1350 SVA putative somatic de novo insertions in total in the three individuals which were present in one brain region but absent in the other and not previously identified as a germline variant.^49^ Thirty-three of these potential de novo insertions were chosen for validation by genotyping PCR and capillary sequencing of the resulting PCR products, successfully validating 28 of them as somatic de novo insertions that were absent from the second brain region.^49^ The number of neurons affected by an individual somatic de novo insertion would be dependent on the point in time of the retrotransposition event, whether it occurred in a single mature postmitotic neuron, during neurogenesis or early in embryonic development affecting neuronal lineages. Therefore, characterising the extent of this neuronal mosaicism is challenging.^26^

Single cell-based analysis of somatic retrotransposition events suggests retrotransposition frequencies of 0.2–16.3 new insertions per neuron depending on the brain region and type of analysis.^50–52^ Although the actual rate of somatic retrotransposition in neurons is still an area of controversy, somatic insertions have been validated and characterised, showing that the genome of an individual or groups of neurons can be altered by these elements. Even taking the lowest rate of 0.2 per neuron would result in 20 billion unique insertions in the adult human brain.^53^ In the adult brain, somatic insertions are more likely to occur into expressed genes due to their open chromatin. It is hypothesised that a controlled level of somatic retrotransposition in neuronal genomes may be beneficial in generating diversity between neurons, thus enabling individuals to respond to their environment, and could be a mechanism involved in memory and learning.^27 49^ Conversely, increased non-LTR retrotransposon activation beyond what may be beneficial and the misregulation of these mobile elements could contribute to neurodegeneration and cognitive decline. Changes to the chromatin structure and derepression of retrotransposons during ageing have been associated with their increased transcript expression. This increase in retrotransposon mRNA expression and evidence for mobilisation has been identified for non-LTR retrotransposons in replicatively senescent human cells (normal diploid fibroblasts), both LTR and non LTR retrotransposons in the ageing mouse (liver and muscle tissue), and LINE-like and LTR retrotransposons in the brain of *Drosophila* where it was linked to cognitive decline.^54–56^ Age is a risk factor for many neurodegenerative conditions, and changes to retrotransposon regulation during ageing may be part of this increasing risk for disease. Although mRNA and retrotransposon encoded proteins have been shown to be upregulated in ALS, it has yet to be determined if there is an increase in somatic retrotransposition events in the brains of patients with ALS and whether the site of insertion could affect the normal functioning of those neurons.

## Summary

ALS is thought to be a multistep process involving a complex interplay between germline genetic variation that defines the level of risk an individual is born with and subsequent modifiers we are confronted with as we age, such as the environment, which determines whether an individual develops the disease or not. The presence or absence of retrotransposon insertions contributes extensively to germline genetic variation affecting the structure, splicing and expression of genes. To date, this type of genetic variation has not yet been addressed in ALS cohorts and should not be overlooked as a potential source of germline risk to the disease. The regulation of these retrotransposons is modified by age and in neurological disorders, including ALS, and can alter the genome of individual or groups of neurons in the adult brain. The changes in the regulation of retrotransposon insertions and the consequences of genomic alterations caused by somatic retrotransposon mobilisation could affect normal cellular function and even lead to cell death, contributing to later steps of the disease development process. Further studies are required to fully investigate the exact role of retrotransposons and determine if there is a direct cause and effect relationship between retrotransposon activity or individual retrotransposon insertions and neurological diseases. Retrotransposons have the potential to be involved in the multiple steps of the development of ALS, from contributing to the missing heritability of the disease to neuronal dysfunction and degenerative processes. Additional references can be found in online supplementary file 1.

10.1136/jnnp-2018-319210.supp1 Supplementary data
