# Supplementary material for: Retrotransposons in the development and progression of amyotrophic lateral sclerosis
Source: J Neurol Neurosurg Psychiatry. 2018 Oct 10;90(3):284–93. doi: 10.1136/jnnp-2018-319210 (PMC6518469; doi:10.1136/jnnp-2018-319210)
Supplement: Supplementary data [file jnnp-2018-319210supp001.pdf]

SUPPLEMENTARY REFERENCES

S1. Al-Chalabi A. Perspective: Don't keep it in the family. Nature 2017;**550**(7676):S112.
S2. Byrne S, Bede P, Elamin M, et al. Proposed criteria for familial amyotrophic lateral sclerosis. Amyotroph Lateral Scler 2011;**12**(3):157-9.
S3. Al-Chalabi A, Fang F, Hanby MF, et al. An estimate of amyotrophic lateral sclerosis heritability using twin data. Journal of neurology, neurosurgery, and psychiatry 2010;**81**(12):1324-6.
S4. Hanby MF, Scott KM, Scotton W, et al. The risk to relatives of patients with sporadic amyotrophic lateral sclerosis. Brain : a journal of neurology 2011;**134**(Pt 12):3454-7.
S5. Al-Chalabi A, van den Berg LH, Veldink J. Gene discovery in amyotrophic lateral sclerosis: implications for clinical management. Nature reviews Neurology 2017;**13**(2):96-104.
S6. Goodier JL, Kazazian HH, Jr. Retrotransposons revisited: the restraint and rehabilitation of parasites. Cell 2008;**135**(1):23-35.
S7. Richardson JM, Dawson A, O'Hagan N, et al. Mechanism of Mos1 transposition: insights from structural analysis. The EMBO journal 2006;**25**(6):1324-34.
S8. Mitra R, Li X, Kapusta A, et al. Functional characterization of piggyBat from the bat Myotis lucifugus unveils an active mammalian DNA transposon. Proceedings of the National Academy of Sciences of the United States of America 2013;**110**(1):234-9.
S9. Thompson PJ, Macfarlan TS, Lorincz MC. Long Terminal Repeats: From Parasitic Elements to Building Blocks of the Transcriptional Regulatory Repertoire. Molecular cell 2016;**62**(5):766-76.
S10. Cohen CJ, Lock WM, Mager DL. Endogenous retroviral LTRs as promoters for human genes: a critical assessment. Gene 2009;**448**(2):105-14.
S11. Barbulescu M, Turner G, Seaman MI, et al. Many human endogenous retrovirus K (HERV-K) proviruses are unique to humans. Current biology : CB 1999;**9**(16):861-8.
S12. Christensen T. Human endogenous retroviruses in neurologic disease. APMIS : acta pathologica, microbiologica, et immunologica Scandinavica 2016;**124**(1-2):116-26.
S13. Khazina E, Weichenrieder O. Non-LTR retrotransposons encode noncanonical RRM domains in their first open reading frame. Proceedings of the National Academy of Sciences of the United States of America 2009;**106**(3):731-6.
S14. Feng Q, Moran JV, Kazazian HH, Jr., et al. Human L1 retrotransposon encodes a conserved endonuclease required for retrotransposition. Cell 1996;**87**(5):905-16.
S15. Babushok DV, Kazazian HH, Jr. Progress in understanding the biology of the human mutagen LINE-1. Human mutation 2007;**28**(6):527-39.
S16. Macia A, Munoz-Lopez M, Cortes JL, et al. Epigenetic control of retrotransposon expression in human embryonic stem cells. Mol Cell Biol 2011;**31**(2):300-16.
S17. Denli AM, Narvaiza I, Kerman BE, et al. Primate-specific ORF0 contributes to retrotransposon-mediated diversity. Cell 2015;**163**(3):583-93.
S18. Burns KH. Transposable elements in cancer. Nat Rev Cancer 2017;**17**(7):415-24.
S19. Ferrante P, Westarp ME, Mancuso R, et al. HTLV tax-rex DNA and antibodies in idiopathic amyotrophic lateral sclerosis. Journal of the neurological sciences 1995;**129 Suppl**:140-4.
S20. Gardner MB, Henderson BE, Officer JE, et al. A spontaneous lower motor neuron disease apparently caused by indigenous type-C RNA virus in wild mice. Journal of the National Cancer Institute 1973;**51**(4):1243-54.
S21. Josephs KA, Whitwell JL, Weigand SD, et al. TDP-43 is a key player in the clinical features associated with Alzheimer's disease. Acta Neuropathol 2014;**127**(6):811-24.
S22. Mackenzie IR, Bigio EH, Ince PG, et al. Pathological TDP-43 distinguishes sporadic amyotrophic lateral sclerosis from amyotrophic lateral sclerosis with SOD1 mutations. Annals of neurology 2007;**61**(5):427-34.
S23. Gotzl JK, Lang CM, Haass C, et al. Impaired protein degradation in FTLD and related disorders. Ageing Res Rev 2016;**32**:122-39.

S24. Buratti E, Baralle FE. TDP-43: gumming up neurons through protein-protein and protein-RNA interactions. Trends in biochemical sciences 2012;**37**(6):237-47.
S25. Muotri AR, Marchetto MC, Coufal NG, et al. L1 retrotransposition in neurons is modulated by MeCP2. Nature 2010;**468**(7322):443-6.
S26. Coufal NG, Garcia-Perez JL, Peng GE, et al. Ataxia telangiectasia mutated (ATM) modulates long interspersed element-1 (L1) retrotransposition in human neural stem cells. Proceedings of the National Academy of Sciences of the United States of America 2011;**108**(51):20382-7.
S27. Shpyleva S, Melnyk S, Pavliv O, et al. Overexpression of LINE-1 Retrotransposons in Autism Brain. Molecular neurobiology 2017.
S28. Bundo M, Toyoshima M, Okada Y, et al. Increased l1 retrotransposition in the neuronal genome in schizophrenia. Neuron 2014;**81**(2):306-13.
S29. Doyle GA, Crist RC, Karatas ET, et al. Analysis of LINE-1 Elements in DNA from Postmortem Brains of Individuals with Schizophrenia. Neuropsychopharmacology : official publication of the American College of Neuropsychopharmacology 2017.
S30. van der Kuyl AC. HIV infection and HERV expression: a review. Retrovirology 2012;**9**:6.
S31. McLaughlin RL, Schijven D, van Rheenen W, et al. Genetic correlation between amyotrophic lateral sclerosis and schizophrenia. Nature communications 2017;**8**:14774.
S32. Sudmant PH, Rausch T, Gardner EJ, et al. An integrated map of structural variation in 2,504 human genomes. Nature 2015;**526**(7571):75-81.
S33. Pace JK, 2nd, Feschotte C. The evolutionary history of human DNA transposons: evidence for intense activity in the primate lineage. Genome research 2007;**17**(4):422-32.
S34. Dupressoir A, Lavialle C, Heidmann T. From ancestral infectious retroviruses to bona fide cellular genes: role of the captured syncytins in placentation. Placenta 2012;**33**(9):663-71.
S35. Beck CR, Garcia-Perez JL, Badge RM, et al. LINE-1 elements in structural variation and disease. Annual review of genomics and human genetics 2011;**12**:187-215.
S36. Zabolotneva AA, Bantysh O, Suntsova MV, et al. Transcriptional regulation of human-specific SVAF(1) retrotransposons by cis-regulatory MAST2 sequences. Gene 2012;**505**(1):128-36.
S37. Faulkner GJ, Kimura Y, Daub CO, et al. The regulated retrotransposon transcriptome of mammalian cells. Nature genetics 2009;**41**(5):563-71.
S38. Kim DS, Hahn Y. Identification of human-specific transcript variants induced by DNA insertions in the human genome. Bioinformatics 2011;**27**(1):14-21.
S39. Sun X, Wang X, Tang Z, et al. Transcription factor profiling reveals molecular choreography and key regulators of human retrotransposon expression. Proceedings of the National Academy of Sciences of the United States of America 2018;**115**(24):E5526-E35.
S40. Hasler J, Strub K. Alu elements as regulators of gene expression. Nucleic acids research 2006;**34**(19):5491-7.
S41. Han JS, Szak ST, Boeke JD. Transcriptional disruption by the L1 retrotransposon and implications for mammalian transcriptomes. Nature 2004;**429**(6989):268-74.
S42. Wheelan SJ, Aizawa Y, Han JS, et al. Gene-breaking: a new paradigm for human retrotransposon-mediated gene evolution. Genome research 2005;**15**(8):1073-8.
